# The S100 protein family in bladder cancer: mechanisms, clinical value, and targeted therapeutic prospects

**DOI:** 10.3389/fonc.2025.1683039

**Published:** 2025-12-01

**Authors:** Lihao Zhang, Gang Yang, Lige Huang, Aijia Deng, Mengxin Ao, Jiabing Li

**Affiliations:** 1 North Sichuan Medical College, Nanchong, China; 2 Department of Urology, Anzhou District People’s Hospital of Miangyang City, Mianyang, China; 3 Southwest Medical University, Luzhou, China; 4 Mianyang Maternal and Child Health Hospital, Mianyang, China; 5 Department of Urology, Sichuan Mianyang 404 Hospital, Mianyang, China

**Keywords:** S100 protein, bladder cancer, immune evasion, rage, epithelial-mesenchymal transition, biomarker, targeted therapy

## Abstract

**Background:**

Bladder cancer (BC) is a highly heterogeneous malignancy with limited molecular biomarkers and therapeutic targets. The S100 protein family, a group of calcium-binding proteins, has emerged as a crucial regulator in cancer development. However, their mechanistic roles and clinical significance in BC remain underexplored.

**Methods:**

This review summarizes the current understanding of the expression patterns, biological functions, and signaling mechanisms of key S100 family members in BC, integrating data from transcriptomic studies, public databases (The Cancer Genome Atlas Program, Gene Expression Omnibus), and recent preclinical research.

**Results:**

S100 family members such as S100A8, S100A9, S100A13, and S100A6 are upregulated in advanced BC and are associated with tumor progression, immune suppression, and poor prognosis. In contrast, S100C exhibits tumor-suppressive properties. Mechanistically, S100 proteins promote epithelial-mesenchymal transition, angiogenesis, and immune evasion by activating receptor for advanced glycation end products(RAGE) and toll-like receptor 4 (TLR4)-mediated signaling pathways. Emerging evidence supports the development of S100-targeted therapeutics including small molecules, monoclonal antibodies, and RAGE inhibitors.

**Conclusion:**

S100 proteins represent promising biomarkers and therapeutic targets in BC. Integrating S100-based profiling into clinical practice may improve molecular classification, prognostication, and personalized treatment. Future efforts should focus on resolving protein redundancy, validating context-specific functions, and advancing drug development for clinical translation.

## 1 Introduction

### 1.1 Structure, classification, and biological functions of S100 proteins

The S100 protein family comprises a group of small calcium-binding proteins characterized by the presence of at least one EF-hand Ca^2+^-binding motif. In humans, this family includes 21 to 22 members, which exhibit high structural similarity yet possess distinct, non-interchangeable functions (1). Most S100 genes are clustered on chromosome 1q21, a genomic region frequently rearranged in various cancers (2). Structurally, S100 proteins function as homodimers or heterodimers, with each monomer containing two EF-hand motifs that undergo conformational changes upon calcium binding—an essential mechanism for their interaction with downstream targets (3). Functionally, they act both intracellularly and extracellularly. Intracellularly, S100 proteins regulate protein phosphorylation, modulate enzymatic activities (e.g., ATPase, adenylate cyclase), influence cytoskeletal dynamics, and affect transcription factor activity (3). Extracellularly, several S100 proteins function as signaling molecules via receptors such as RAGE and TLR4, thereby modulating inflammation, proliferation, apoptosis, and migration (1, 4). Under physiological conditions, S100 protein expression is tightly regulated in a cell- and tissue-specific manner. Epigenetic modifications, such as promoter deoxyribonucleic acid(DNA) methylation, play critical roles in controlling their expression profiles (5). Dysregulation of S100 expression is common in malignancies, contributing to tumor progression, metastasis, and prognosis (6). For example, S100A8 and S100A9 activate mitogen-activated protein kinases(MAPK) and nuclear factor-kappaB(NF-κB) signaling, promoting migration and inflammation in cancer cells (7). S100A4 is also well-established as a driver of metastasis and epithelial–mesenchymal transition(EMT)through interactions with matrix metalloproteinases (8, 9). Due to their multifaceted biological roles, S100 proteins are increasingly recognized as potential diagnostic and prognostic biomarkers, as well as therapeutic targets. Drug development efforts have yielded inhibitors targeting specific S100 members, with some entering clinical trials (1, 10). Understanding the individual contributions of S100 proteins in cancer—particularly BC—is crucial to harnessing their clinical utility.

### 1.2 Epidemiology and molecular pathogenesis of BC

BC is one of the most prevalent malignancies of the urinary tract, contributing significantly to global cancer morbidity and mortality (11). It demonstrates notable heterogeneity in histological subtypes, molecular characteristics, and clinical behavior. Clinically, BC is broadly classified into non-muscle-invasive bladder cancer (NMIBC) and muscle-invasive bladder cancer (MIBC), each with distinct molecular features and clinical courses (12, 13). Epidemiologically, several environmental and lifestyle factors contribute to BC risk. Tobacco smoking is the most established risk factor, accounting for approximately half of all cases (14). Occupational exposure to aromatic amines, arsenic in drinking water, and dietary influences also play roles. Genetic predisposition is increasingly recognized, with familial clustering observed in epidemiological studies. Individuals with a first-degree relative diagnosed with BC face nearly a twofold increased risk, independent of shared environmental exposures such as smoking (15). Molecular studies, including genome-wide association studies (GWAS) and next-generation sequencing, have identified numerous genetic alterations associated with BC development and progression. These include mutations in the promoter region of the telomerase reverse transcriptase gene (TERT), and alterations in key oncogenes and tumor suppressor genes such as FGFR3, TP53, and PIK3CA, as well as genes involved in chromatin remodeling (16–18). BC evolves through at least two distinct oncogenic pathways. The papillary/luminal pathway is commonly associated with FGFR3 mutations and characterizes NMIBC. In contrast, the non-papillary/basal pathway is frequently driven by TP53 mutations and is linked to MIBC (19). These divergent pathways correspond to intrinsic molecular subtypes—luminal and basal—that possess distinct gene expression profiles with implications for prognosis and treatment selection (13). Notably, the mutational burden is generally higher in MIBC than in NMIBC (11). Advances in proteomic and transcriptomic profiling have further clarified the molecular landscape of BC. Dysregulated biological processes include extracellular matrix organization, oxidative stress response, RNA splicing, and macromolecular complex assembly (20, 21). Gene set variation analysis has revealed enrichment of critical signaling pathways such as Notch, Wnt/β-catenin, apoptosis, and coagulation cascades in bladder tumorigenesis (22). These molecular insights have facilitated the discovery of candidate biomarkers and therapeutic targets, paving the way for improved early diagnosis, prognostic stratification, and personalized treatment strategies (23, 24).

### 1.3 Expression profiles of S100 proteins in BC

The altered expression of S100 family members in BC has garnered increasing attention due to their implications for tumor biology and clinical outcomes. Several S100 proteins show differential expression in BC tissues compared to normal urothelium, suggesting their potential roles in tumor progression, metastasis, and prognosis.S100C (also known as S100A10) is notably downregulated during BC progression. Comparative proteomic analyses have demonstrated significantly reduced S100C mRNA levels in invasive tumors (T1 and T2–T4 stages) relative to superficial tumors (Ta stage), with a strong inverse correlation to histological grade. Lower S100C expression is significantly associated with poorer overall survival, underscoring its potential as a prognostic biomarker (25). In contrast, other S100 proteins such as S100A6, S100A8, S100A9, and S100A11 are frequently upregulated in various malignancies, including BC, where they are linked to tumor-promoting functions (26, 27). For instance, increased expression of S100A8 and S100A9 is associated with pro-inflammatory signaling and enhanced tumor cell migration. A particularly notable BC-specific nuclear protein, BLCA-1, has been identified as a potential urinary biomarker. Detected in both tissue and urine samples from BC patients—but absent in normal bladder tissue—BLCA-1 showed 80% sensitivity and 87% specificity for BC detection, independent of tumor grade (28). These findings highlight the potential for S100-related proteins to serve as non-invasive diagnostic markers. Proteomic profiling using tissue microarrays and mass spectrometry has further confirmed the altered expression of S100 proteins in BC. For example, high levels of S100A6 and other immunogenic proteins have been found to correlate with histological grade and overall survival, particularly in MIBC (29). Integrative analyses combining proteomic and transcriptomic datasets have revealed that S100 family members are involved in biological processes related to proliferation, migration, and immune modulation within the tumor microenvironment (20). Together, these findings illustrate the diverse, context-dependent roles of S100 proteins in BC. Their differential expression patterns not only reflect tumor behavior but also provide promising opportunities for the development of diagnostic, prognostic, and therapeutic strategies.

## 2 Molecular mechanisms of S100 proteins in BC development

### 2.1 Regulation of cell proliferation, apoptosis, and cell cycle

Members of the S100 protein family play pivotal roles in regulating critical cellular processes involved in BC development, including proliferation, apoptosis, and cell cycle progression. Acting as both intracellular calcium sensors and extracellular signaling mediators, S100 proteins influence tumorigenic pathways in a highly context-dependent manner (1). Several S100 proteins have been implicated in the dysregulation of proliferation in various cancers. For example, S100P has been shown to promote cancer cell growth by interacting with key regulators. In colon cancer models, lentivirus-mediated knockdown of S100P significantly reduced tumor proliferation and metastasis, suggesting an oncogenic role (30). Mechanistically, S100P modulates downstream effectors such as thioredoxin 1 and β-tubulin, which are involved in redox balance and cytoskeletal integrity, respectively (30). In pancreatic cancer, overexpression of S100P enhances proliferation through cytoskeletal reorganization and upregulation of invasion-associated proteins such as cathepsin D (31). S100P also interacts with tumor suppressor pathways. It binds to both p53 and its negative regulator HDM2, disrupting their interaction and stabilizing p53. However, this stabilized p53 is functionally impaired, lacking phosphorylation and failing to activate its transcriptional targets (e.g., p21, Bax), thereby reducing apoptosis and promoting therapy-induced senescence (32). This mechanism contributes to chemoresistance and tumor progression. Epigenetic modifications further modulate S100 protein expression and function. In addition to transcriptional and epigenetic regulation, post-translational modifications (PTMs) critically influence S100 protein stability and activity (33). Various PTMs—including phosphorylation, oxidation, acetylation, and S-nitrosylation—modulate the conformational dynamics of S100 proteins, thereby altering their affinity for calcium ions, target proteins, and membrane receptors (33, 34). For instance, phosphorylation of S100A4 and S100A9 can enhance their interaction with cytoskeletal and inflammatory signaling molecules, promoting motility and NF-κB activation (32, 35). Oxidation of S100A8/A9 alters their dimerization status and inflammatory potential, functioning as a molecular switch between pro-tumor and anti-tumor states under oxidative stress conditions (36). Moreover, acetylation may affect nuclear localization and transcriptional regulatory capacity of certain S100 members (33). These PTM-mediated alterations underscore the fine-tuned molecular plasticity of S100 proteins and may partly explain their context-dependent roles in bladder cancer progression and therapy response. Aberrant DNA methylation in the regulatory regions of S100 genes can lead to dysregulated expression, contributing to altered cell cycle progression and evasion of apoptosis in cancer cells (5). Other family members, such as S100A11, also act as oncogenic drivers. In gastric cancer, S100A11 overexpression correlates with poor prognosis and promotes metastasis through activation of matrix metalloproteinases and EMT. Silencing S100A11 suppresses metastasis and enhances chemosensitivity to agents like 5-fluorouracil and cisplatin (37). Collectively, these findings illustrate that S100 proteins regulate BC cell behavior by modulating signaling pathways associated with proliferation, apoptosis, and the cell cycle. Their dysregulation promotes tumor progression and therapy resistance, highlighting their potential as targets for anticancer intervention. Future proteomic studies focusing on PTM profiling of S100 proteins in bladder cancer tissues may provide valuable insights into dynamic regulation and therapeutic vulnerabilities.

### 2.2 Roles in tumor invasion, metastasis, and angiogenesis

S100 proteins play critical roles in BC progression by promoting tumor invasion, metastasis, and, potentially, angiogenesis. Their functions in these processes involve modulation of cytoskeletal dynamics, interaction with extracellular matrix (ECM) components, and activation of pro-metastatic signaling pathways.S100A4 is one of the best-characterized S100 family members implicated in metastasis. Elevated S100A4 expression is associated with enhanced tumor motility, invasion, and poor prognosis across multiple cancer types, including BC (8, 9). Mechanistically, S100A4 promotes EMT and interacts with matrix metalloproteinases (MMPs), facilitating ECM degradation and enabling tumor cell dissemination (6). S100P also contributes significantly to tumor invasion and metastasis. Its overexpression results in cytoskeletal reorganization, characterized by actin filament disassembly and altered phosphorylation of actin-regulating proteins such as cofilin. These changes enhance cell motility and invasive potential (31). Furthermore, S100P upregulates cathepsin D, a lysosomal protease involved in ECM remodeling and metastatic spread. In colon cancer models, S100P knockdown reduces both migration and liver metastasis, highlighting its role in cancer dissemination (30). Although direct evidence for S100-mediated angiogenesis in BC remains limited, studies in other cancers suggest a likely role. S100 proteins, particularly S100A8 and S100A9, have been shown to modulate the tumor microenvironment in ways that promote neovascularization, thereby supporting tumor expansion and metastasis (38). These pro-angiogenic effects may involve interactions with endothelial cells, inflammatory mediators, and VEGF-related pathways. Interestingly, the metastatic behavior associated with certain S100 proteins can be context-dependent. For example, in breast cancer, high S100B expression is associated with reduced migratory capacity and improved prognosis, indicating that not all S100 members universally promote metastasis (39). In summary, S100 proteins facilitate BC progression by orchestrating key events in invasion, metastasis, and potentially angiogenesis. Their expression patterns correlate with aggressive tumor phenotypes, underscoring their value as both prognostic markers and therapeutic targets in advanced BC.

### 2.3 Modulation of the tumor microenvironment and immune response

**Figure 1:**
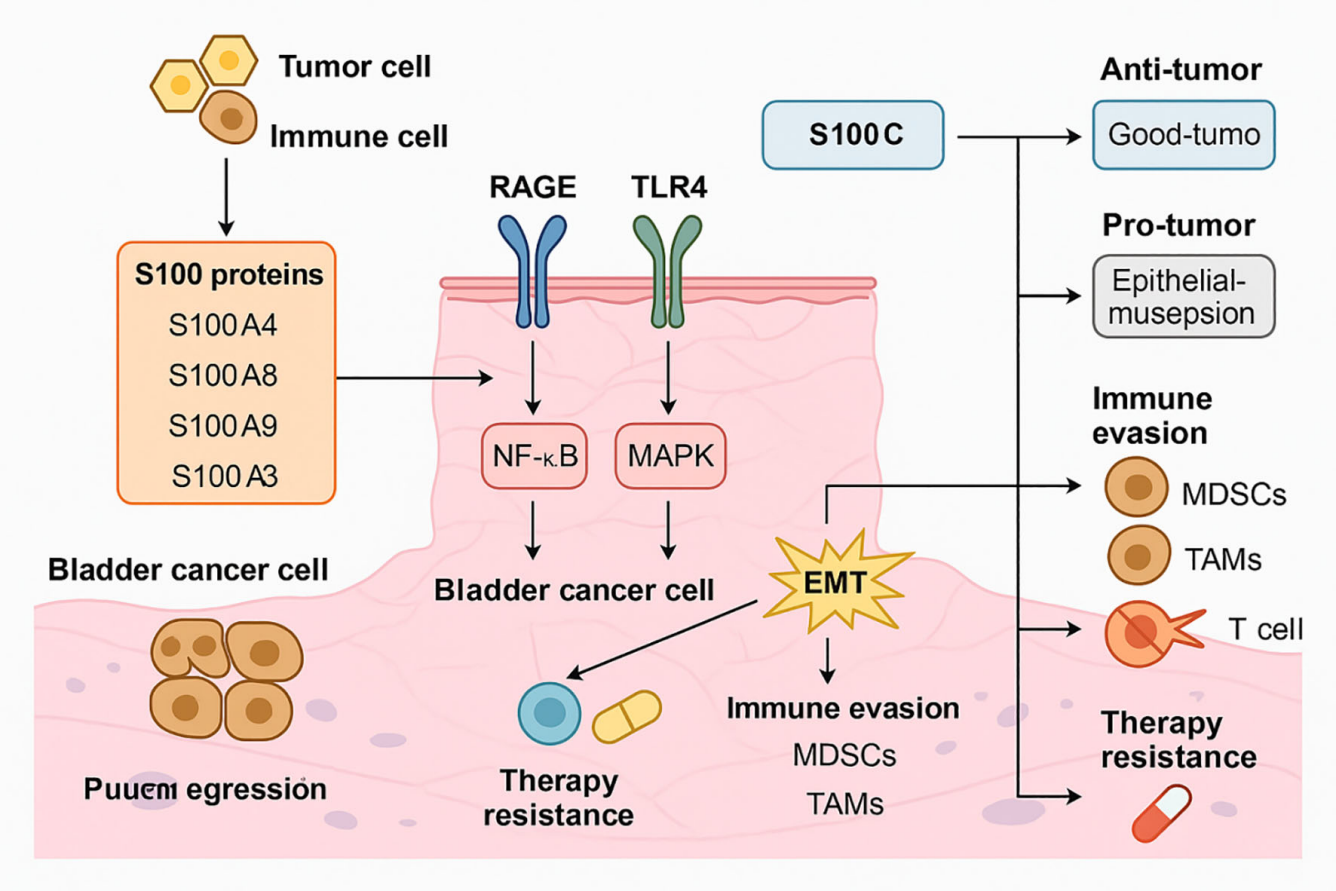
Mechanistic roles of S100 family proteins in BC progression, immune evasion, and therapy resistance. S100A4, S100A8, S100A9, and S100A3 are secreted by tumor and immune cells within the BC microenvironment. These proteins interact with RAGE and TLR4 receptors on BC cells, triggering downstream activation of the NF-κB and MAPK pathways. This signaling cascade promotes EMT, which enhances tumor cell invasiveness and facilitates escape from immune surveillance. In parallel, S100-mediated signaling leads to the recruitment of immunosuppressive cells such as myeloid-derived suppressor cells (MDSCs) and tumor-associated macrophages (TAMs), further contributing to immune evasion and dampening of T cell-mediated responses. These processes synergistically result in enhanced therapy resistance. Additionally, S100C exerts dual, context-dependent effects: in some settings it supports anti-tumor immunity, while in others it promotes epithelial disruption and tumor progression. The combined effects of S100 proteins on signaling activation, immune modulation, and treatment resistance highlight their central role in BC biology and support their value as potential diagnostic and therapeutic targets.

S100 proteins significantly influence the tumor microenvironment (TME) and immune responses in BC, contributing to tumor progression and therapeutic resistance. These effects are mediated through interactions with immune cells, modulation of inflammatory signaling, and facilitation of immune evasion. The TME in BC comprises tumor cells, stromal cells, immune infiltrates, and extracellular matrix components. S100 proteins function as damage-associated molecular patterns (DAMPs), activating innate immune receptors such as RAGE and TLR4, thereby initiating pro-inflammatory cascades (1). S100A8 and S100A9, for instance, activate the MAPK and NF-κB pathways, which in turn promote cytokine production, leukocyte recruitment, and tumor-associated inflammation (7). S100P is increasingly recognized as a key immunomodulator. In pancreatic cancer, its expression correlates with an immunosuppressive microenvironment, characterized by reduced CD8^+^ T cell infiltration and increased expression of immune checkpoint molecules, including TIGIT, CTLA-4, and BTLA (40). Although such associations have not been fully validated in BC, these findings suggest that S100P may play a similar role in shaping an immune-evasive TME.BC is known for its high recurrence and immune escape potential. Immunosuppressive populations, such as regulatory T cells (Tregs), are often enriched within the TME. An inverted ratio of effector T cells to Tregs has been correlated with disease recurrence, underscoring the critical role of immune imbalance in tumor persistence (41). S100 proteins, through their ability to modulate inflammatory cytokines and immune checkpoints, may contribute to this imbalance. Proteomic analyses of BC have identified biomarkers related to immune infiltration and prognosis. For example, chloride intracellular channel protein 1 (CLIC1), although not an S100 member, has been associated with immune cell infiltration and adverse outcomes (42). Furthermore, systemic inflammatory markers such as C-reactive protein (CRP) have been linked to poor survival, suggesting that inflammation-related proteins, including S100 family members, may serve as prognostic indicators (43). Emerging evidence indicates that S100 proteins may also mediate resistance to immunotherapy. Their roles in modulating immune checkpoints and maintaining an immunosuppressive TME suggest that S100-targeted strategies could enhance the efficacy of immune checkpoint blockade (44). In summary, S100 proteins actively shape the immune landscape of BC by regulating inflammatory signaling, immune cell infiltration, and checkpoint expression. These functions support tumor immune evasion and may limit the effectiveness of current immunotherapies, making S100 proteins attractive candidates for combination strategies aimed at overcoming resistance. The functional complexity of S100 family members in BC is further illustrated in **Figure 1**, which outlines the major mechanisms by which these proteins contribute to tumor progression, immune evasion, and therapy resistance.

Beyond immune modulation, S100 proteins exert profound effects on other cellular components of the tumor microenvironment. Cancer-associated fibroblasts (CAFs) respond to extracellular S100A4, S100A8, and S100A9 by activating pro-inflammatory and pro-fibrotic signaling pathways, which enhance extracellular matrix remodeling and facilitate tumor invasion (45). Endothelial cells exposed to S100A13 and S100A6 display increased angiogenic activity through VEGF and ERK pathway activation, linking S100 signaling to neovascularization and nutrient supply within the tumor (46). Moreover, tumor-associated macrophages (TAMs) and myeloid-derived suppressor cells (MDSCs) are recruited and polarized by S100A8/A9 and S100P, reinforcing an immunosuppressive milieu that limits cytotoxic T cell infiltration (40, 43). These interactions collectively establish a feed-forward loop between S100 proteins and the TME, promoting tumor progression, metastasis, and resistance to immunotherapy (44). Targeting these S100-mediated stromal and immune interactions may therefore represent a promising avenue for combined therapeutic strategies in bladder cancer (50).

## 3 Clinical relevance of S100 proteins in BC

### 3.1 Diagnostic and prognostic biomarker value

The S100 protein family, characterized by conserved calcium-binding EF-hand motifs, has gained increasing attention for its diagnostic and prognostic utility across various malignancies, including BC. Aberrant expression of S100 proteins is frequently observed in cancer tissues, and specific expression patterns often correlate with tumor progression, grade, and patient outcomes (1). In BC, several S100 proteins have emerged as potential biomarkers. For example, S100C (also referred to as S100A10) is significantly downregulated in invasive bladder tumors. Quantitative PCR analysis of 88 BC specimens showed markedly reduced S100C mRNA levels in muscle-invasive tumors (T2–T4) compared to superficial tumors (Ta). Furthermore, low S100C expression was associated with higher tumor grade and worse overall survival, indicating its potential as a tumor suppressor and prognostic marker (25). S100A8 has also shown clinical relevance. A study analyzing 103 primary NMIBC specimens identified an S100A8-correlated gene expression signature that predicted disease progression. This signature yielded a hazard ratio of 15.225 for progression, and its predictive capacity was validated in an independent cohort of 302 patients (47). These results suggest that S100A8 may help identify NMIBC patients at high risk of progressing to MIBC. Urinary biomarkers based on S100-related proteins have also been explored. BLCA-1, a BC-specific nuclear structural protein, was detectable in both tumor tissues and urine samples, but not in normal bladder tissues. The urinary test demonstrated a sensitivity of 80% and specificity of 87%, independent of tumor grade (28). This highlights the potential of S100-associated proteins for non-invasive detection and monitoring of BC. Large-scale proteomic studies have supported the inclusion of S100 proteins in multi-marker panels. For instance, S100 family members have been identified as differentially expressed in tumors using mass spectrometry, correlating with stage and grade (20). Circulating proteomic signatures involving S100 proteins have also been proposed for early diagnosis and risk stratification (48). In summary, S100 proteins—through their altered expression in tumor tissues and bodily fluids—represent promising biomarkers for BC diagnosis and prognosis. Further validation in large, multi-center clinical studies and standardization of detection assays are essential for their translation into routine clinical practice.

### 3.2 S100 protein levels in non-invasive versus muscle-invasive disease

The expression levels of S100 proteins vary significantly between NMIBC and MIBC, reflecting their involvement in tumor progression and aggressiveness. Proteomic and transcriptomic studies have demonstrated distinct S100 expression patterns corresponding to disease stage.S100C expression is notably decreased in MIBC compared to NMIBC. Quantitative analyses have revealed that S100C mRNA levels are significantly reduced in T1 and muscle-invasive tumors (T2–T4) relative to superficial tumors (Ta), with a strong negative correlation to histopathological grade (25). This downregulation is evident early in disease development and is associated with poor clinical outcomes, suggesting that S100C loss may contribute to invasive phenotypes. Conversely, S100A8 and S100A9 expression is elevated in more advanced stages of BC. In a study comparing MIBC and NMIBC tissues, these proteins were significantly upregulated in invasive tumors, implicating them in disease progression (47). These findings are supported by their known roles in promoting inflammation, migration, and immune modulation. Additional proteomic analyses have identified S100 proteins among broader panels of differentially expressed proteins in BC subtypes. For example, mass spectrometry of BC serum and tissue samples has revealed elevated expression of proteins, including S100 family members, associated with tumor stage, grade, and prognosis (42). Gene expression-based molecular classification has further highlighted the stage-specific roles of S100 proteins. In particular, transcriptomic subtyping has improved prognostic stratification in T1 tumors, where clinical outcomes are highly variable. S100 expression patterns have contributed to defining molecular subgroups with distinct clinical behavior and therapeutic response profiles (49). Collectively, these findings suggest that S100 proteins not only serve as indicators of tumor stage but may actively drive the transition from non-invasive to invasive disease. Their differential expression patterns may be leveraged for risk stratification and treatment planning in BC.

The functional heterogeneity of the S100 family arises from multiple layers of molecular regulation. Although structurally homologous, individual S100 proteins differ in subcellular localization, PTMs, binding partners, and receptor affinity, all of which dictate their downstream biological consequences. For instance, S100A8 and S100A9, when secreted as calprotectin, act as damage-associated molecular patterns (DAMPs) that engage RAGE or TLR4, triggering pro-inflammatory and pro-tumorigenic cascades such as NF-κB and STAT3 activation (50). In contrast, S100C (S100A10) primarily functions intracellularly in association with annexin 2, modulating cytoskeletal stability and suppressing cell motility, which confers tumor-suppressive potential (51). Moreover, oxidative stress–dependent PTMs can alter S100 conformations and switch their receptor interactions, further contributing to context-dependent duality (52). These differences underscore that the oncogenic or tumor-suppressive roles of S100 proteins are not intrinsic but rather determined by molecular context, cellular localization, and interaction networks within the bladder tumor microenvironment.

Accumulating evidence indicates that S100 family members display distinct expression patterns across molecular subtypes and pathological stages of bladder cancer, reflecting their diverse biological functions. Basal-like tumors, characterized by high epithelial–mesenchymal transition (EMT) activity, immune infiltration, and poorer clinical outcomes, exhibit elevated levels of S100A8, S100A9, and S100A14 (53). These proteins are known to amplify inflammatory signaling through the RAGE/TLR4–NF-κB axis, thereby promoting tumor invasion, angiogenesis, and immune evasion (57). In contrast, luminal-type tumors, defined by GATA3, KRT20, and uroplakin expression, tend to exhibit higher expression of differentiation-associated S100 members such as S100A1 and S100C (S100A10), which are enriched in terminally differentiated urothelial cells and may contribute to epithelial stability and a less aggressive phenotype (54). From a disease stage perspective, several studies have shown that S100A4 and S100A8/A9 are significantly upregulated in muscle-invasive bladder cancer (MIBC) compared with non–muscle-invasive bladder cancer (NMIBC), supporting their roles in facilitating invasion and metastasis (25). Together, these findings highlight that S100 proteins exhibit subtype- and stage-specific expression profiles in bladder cancer, which may serve as molecular correlates of tumor aggressiveness and potential biomarkers for patient stratification and therapeutic guidance.

### 3.3 Correlation with clinicopathological parameters and patient outcomes

The expression of S100 proteins in BC has been significantly correlated with key clinicopathological features such as tumor stage, grade, lymphovascular invasion, recurrence, and overall survival. These associations highlight the prognostic potential of S100 family members and their value in clinical decision-making.S100C, for example, has been shown to have an inverse relationship with tumor grade and stage. In a study of 88 BC specimens, S100C expression was significantly lower in high-grade and muscle-invasive tumors compared to low-grade and superficial lesions (25). Importantly, patients with low S100C levels had shorter overall survival, supporting its role as an independent prognostic factor.S100A8 and S100A9, by contrast, are often upregulated in aggressive disease and correlate positively with advanced pathological features. High expression of these proteins has been associated with increased risk of recurrence and progression. Their inflammatory roles suggest that they may contribute to tumor-promoting immune microenvironments, further exacerbating disease aggressiveness (47). S100A6 has also been linked to poor prognosis in BC. In proteomic analyses, its elevated expression was associated with high histological grade and reduced survival. These findings support the integration of S100A6 into biomarker panels for outcome prediction (20). Moreover, the correlation between S100 protein levels and survival outcomes has been validated across several datasets, including The Cancer Genome Atlas (TCGA) and Gene Expression Omnibus (GEO). These public resources have demonstrated that patients with dysregulated S100 expression—either upregulation or downregulation depending on the subtype—experience worse disease-specific or overall survival (20, 29). Taken together, these observations reinforce the relevance of S100 proteins in predicting BC behavior and prognosis. Incorporating S100 protein assessment into clinical workflows may enhance individualized risk stratification, inform surveillance schedules, and guide treatment selection.

### 3.4 Limitations and subtype-specific considerations

Despite increasing evidence linking S100 protein expression to bladder cancer stage, grade, and prognosis, most existing studies have not comprehensively accounted for molecular subtype specificity or adjusted for clinical confounders. The expression and prognostic value of S100 proteins can vary substantially among molecular subtypes—for example, S100A7 and S100A9 are frequently upregulated in basal-type tumors, whereas S100A1 and S100B appear more enriched in luminal subtypes (55). These context-dependent differences suggest that subtype-specific expression patterns should be considered when interpreting prognostic associations. Moreover, many published analyses rely on univariate comparisons without correction for variables such as age, tumor stage, treatment modality, and inflammatory status, which may lead to biased estimates. Future studies integrating multivariate modeling and subtype-stratified validation across large, well-annotated clinical cohorts are warranted to refine the predictive and therapeutic implications of S100-related biomarkers. Addressing these analytical limitations will be essential for translating S100-based biomarkers from discovery to clinical application.

## 4 Targeting S100 proteins in BC: therapeutic prospects

### 4.1 Rationale for targeting S100 proteins

The consistent involvement of S100 proteins in tumor growth, invasion, immune evasion, and treatment resistance provides a compelling rationale for developing targeted therapies against this family in BC. Their multifaceted roles in promoting oncogenesis and shaping the tumor microenvironment suggest that S100 proteins may serve not only as biomarkers but also as direct therapeutic targets. Many S100 proteins act as key mediators of oncogenic signaling pathways. For instance, S100P enhances cancer cell proliferation, cytoskeletal reorganization, and resistance to apoptosis, contributing to tumor progression and poor treatment response (30, 31). Similarly, S100A4 and S100A11 promote EMT, invasion, and metastasis by interacting with matrix metalloproteinases and modulating cytoskeletal proteins (6, 37). These properties make them attractive candidates for therapeutic inhibition. Additionally, S100 proteins modulate inflammatory and immune responses, often creating an immunosuppressive microenvironment. This can impair the efficacy of immune checkpoint inhibitors in BC, where immune evasion is a major hurdle to treatment success (44, 44). Targeting S100-mediated pathways could restore immune surveillance and improve responses to immunotherapy. From a practical standpoint, several features make S100 proteins amenable to therapeutic targeting. These include their overexpression in cancer tissues, presence in extracellular compartments, and involvement in ligand-receptor interactions—such as with RAGE and Toll-like receptor 4 (TLR4)—that can be disrupted by small molecules or monoclonal antibodies (1, 7). Moreover, S100 proteins often function extracellularly in an autocrine or paracrine manner, further supporting their drug accessibility. Altogether, these observations provide strong justification for targeting S100 proteins in BC. Therapeutic interventions designed to inhibit S100 signaling may simultaneously suppress tumor proliferation, reduce metastasis, and overcome immune resistance, offering a multipronged approach to improve patient outcomes.

### 4.2 Emerging small molecule and biologic inhibitors of S100 proteins

Advancements in drug development have led to the identification of small molecules and biologics that specifically target S100 proteins or their downstream signaling pathways. These agents have demonstrated antitumor efficacy in preclinical models of various cancers, offering new therapeutic avenues for BC. Trifluoperazine (TFP), a calmodulin antagonist originally used as an antipsychotic, has been shown to inhibit S100A4 function and block tumor cell migration and invasion *in vitro* and *in vivo*. TFP interferes with S100A4-mediated cytoskeletal remodeling and reduces metastatic potential in breast and pancreatic cancer models (56). Although its effects in BC remain unexplored, its ability to target S100A4 makes it a promising candidate for future studies. Amlexanox, an anti-inflammatory and anti-allergic agent, inhibits S100A13-mediated pathways. In thyroid cancer cells, amlexanox downregulates S100A13 expression and reduces cell proliferation and invasion, partly by modulating downstream angiogenic and inflammatory mediators (57). Given the pro-angiogenic role of S100A13 in other malignancies, repurposing amlexanox for BC therapy warrants investigation. Another potential strategy involves targeting the interaction between S100 proteins and their receptors. The receptor for RAGE is a major binding partner for several S100 proteins, including S100P, S100A8, and S100A9. Inhibiting the S100–RAGE axis can disrupt oncogenic signaling cascades such as NF-κB and MAPK pathways. Small molecule RAGE inhibitors and decoy receptors have shown promise in reducing tumor growth and inflammation in animal models (58). Biologic therapies targeting S100 proteins are also being explored. Monoclonal antibodies and peptide inhibitors designed to block S100–receptor interactions have demonstrated anti-proliferative and anti-metastatic effects in preclinical studies. For example, antibodies against S100A9 suppressed tumor growth in prostate cancer models by modulating the immune microenvironment (59). To provide a clearer overview of the current therapeutic landscape, **Table 1** summarizes representative small molecules and biologic agents that target S100 proteins or their downstream signaling pathways, including their mechanisms of action and supporting evidence. Beyond preclinical compounds, several S100-related therapeutic agents have advanced into clinical evaluation, primarily through targeting the S100–RAGE signaling axis. The RAGE antagonist azeliragon (TTP488), initially developed for Alzheimer’s disease, has entered multiple phase II clinical trials evaluating its safety and anti-inflammatory potential in oncology and metabolic disorders (NCT02080364, NCT029160)[ClinicalTrials.gov]. Similarly, the small-molecule RAGE inhibitor FPS-ZM1 has demonstrated favorable pharmacokinetics and tumor-suppressive effects *in vivo*, serving as a lead compound for next-generation derivatives (60). In addition, neutralizing antibodies against S100A8/A9 (e.g., tasquinimod) have shown promising immunomodulatory and anti-angiogenic effects in prostate and colorectal cancer models, supporting the feasibility of S100-targeted immunotherapy (61). Importantly, combination strategies involving S100 pathway inhibition with standard treatments are emerging as a rational approach to overcome therapeutic resistance. For instance, co-targeting S100A8/A9–RAGE signaling with immune checkpoint blockade may alleviate myeloid-derived immune suppression and enhance T-cell activation, while combining S100 inhibition with chemotherapy could mitigate treatment-induced inflammatory feedback that promotes recurrence (62). These mechanistic complementarities suggest that S100-targeted drugs, either as monotherapy or in rational combinations, hold promise for translation into future precision treatment regimens for bladder cancer. Despite these encouraging findings, no S100-targeted therapies have yet reached clinical approval. Challenges include protein redundancy, tissue-specific functions, and compensatory pathways that may reduce efficacy. Nevertheless, the therapeutic potential of S100 inhibition remains significant, particularly in combination with existing treatments.

**Table 1:** Representative small molecules and biologics targeting S100 proteins or their downstream signaling pathways.

Category	Compound/Agent	Primary target (S100 member or pathway)	Mechanism of action	Cancer model/evidence
Small molecule	Trifluoperazine	S100A4	Inhibits S100A4-mediated cytoskeletal remodeling and metastasis	Breast, pancreatic cancer (*in vitro* and *in vivo*)
Small molecule	Amlexanox	S100A13	Downregulates S100A13 and suppresses angiogenic/inflammatory signaling	Thyroid cancer cell models
Small molecule	FPS-ZM1	RAGE (S100A8/A9/P downstream receptor)	Blocks S100–RAGE interaction, inhibits NF-κB activation	Multiple cancer models
Monoclonal antibody	Anti-S100A9 antibody	S100A9	Neutralizes S100A9, reduces tumor growth via immune modulation	Prostate cancer xenografts
Peptide inhibitor	RAGE decoy peptide	S100A8/A9–RAGE	Prevents ligand–receptor binding, suppresses inflammation	Preclinical tumor models
Combination approach	S100 inhibition + immunotherapy	S100P/immune checkpoints	Enhances immune response, overcomes TME-mediated resistance	Preclinical studies, under exploration in BC

### 4.3 Challenges and perspectives in targeting S100 proteins

#### 4.3.1 S100 proteins and therapy resistance

Emerging evidence indicates that S100 family members contribute to resistance to multiple treatment modalities in cancer, and several mechanistic routes are plausible in bladder cancer (63). First, S100P has been reported to bind and functionally inactivate p53, permitting therapy-induced senescence and contributing to chemoresistance in preclinical models; this suggests that S100P upregulation may blunt p53-dependent apoptotic responses to DNA-damaging agents (32). Second, S100A8/A9 promote a pro-inflammatory yet immunosuppressive tumor microenvironment by recruiting and polarizing myeloid populations (e.g., myeloid-derived suppressor cells, Tumor-associated macrophages) and upregulating immune checkpoint molecules, which can impair the efficacy of immune checkpoint blockade (64). Third, S100 proteins such as S100A4 and S100A11 facilitate EMT and cytoskeletal remodeling, phenotypes commonly associated with reduced chemosensitivity and enhanced metastatic seeding (9). Fourth, S100-mediated activation of downstream pro-survival pathways [e.g., NF-κB, MAPK/ERK(extracellular signal-regulated kinase)]can promote cell survival under therapeutic stress and potentially reduce the efficacy of targeted inhibitors (63). **Table 2** summarizes key S100 family members and their reported roles in therapeutic resistance in bladder cancer. Collectively, these mechanisms indicate that S100 proteins may act as both intrinsic and microenvironment-mediated drivers of therapeutic resistance.

**Table 2:** Selected evidence linking S100 proteins to therapy resistance.

Therapy type	S100 member(s)	Suggested mechanism(s)	Evidence/cancer models
Chemotherapy	S100P	p53 inactivation → reduced apoptosis/therapy-induced senescence	*In vitro*/preclinical (cancer models)
Immune checkpoint blockade/Immunotherapy	S100A8/S100A9; S100P	Recruitment/polarization of MDSCs & TAMs; immune suppression; upregulation of checkpoints	Bioinformatics + *in vitro*/preclinical
Chemotherapy/Metastasis-associated resistance	S100A4, S100A11	EMT promotion; cytoskeletal remodeling → reduced chemosensitivity and increased invasion	Preclinical studies (multiple cancers)
Broad (pro-survival signaling)	Various (S100A8/A9, S100P)	Activation of NF-κB/MAPK → cell survival under stress	Proteomic and mechanistic studies

From a translational perspective, these findings support two key implications. (1) S100 expression or activity may serve as a predictive biomarker for response to chemotherapy, immunotherapy, or targeted treatments in BC, and should be evaluated in retrospective cohorts and prospective trials (63, 64). (2) Combination approaches that concurrently inhibit S100 signaling (directly or via shared receptors such as RAGE/TLR4) and standard therapies may overcome resistance and improve outcomes; preclinical studies combining S100 blockade with immunotherapy or chemotherapy warrant prioritization (65). Overall, recognizing S100 proteins as modulators of therapy response could inform patient stratification and the design of rational combination regimens in bladder cancer.

Despite the promising preclinical evidence, several challenges complicate the therapeutic targeting of S100 proteins in BC. The functional redundancy and overlapping expression patterns of S100 family members complicate the identification of the most effective targets. The functional complexity of the S100 family is further compounded by the interconnected and compensatory nature of its members. Many S100 proteins exhibit overlapping ligand-binding properties and converge on common downstream effectors, including RAGE, TLR4, and NF-κB signaling pathways, leading to similar pro-tumorigenic outcomes such as inflammation, angiogenesis, and epithelial–mesenchymal transition (66). For instance, inhibition of S100A4 can be partially compensated by upregulation of S100A6 or S100A11, maintaining cytoskeletal remodeling and invasive potential (67, 68). Similarly, both S100A8 and S100A9 act cooperatively as heterodimers (calprotectin), amplifying inflammatory signaling and promoting immune evasion (69). These overlapping functions underscore the redundant network behavior of S100 proteins in BC, suggesting that targeting a single member may be insufficient to achieve durable therapeutic efficacy (63). Future research should focus on integrated approaches that consider multi-target inhibition or modulation of shared receptors and downstream signaling hubs to overcome this redundancy and achieve synergistic therapeutic benefit. Moreover, the context-dependent roles of these proteins, which can vary between intracellular and extracellular environments, add layers of complexity to drug development (58). The structural similarity among S100 proteins poses difficulties in designing highly selective inhibitors that minimize off-target effects. Additionally, the dynamic interactions of S100 proteins with multiple receptors, such as the receptor for RAGE and TLR4, contribute to diverse signaling outcomes that are not yet fully elucidated (59). This incomplete understanding of molecular mechanisms limits the rational design of targeted therapies. In BC, tumor heterogeneity and lineage plasticity further challenge the efficacy of S100-targeted treatments. The variability in S100 protein expression across different tumor subtypes and stages necessitates precise biomarker-driven patient stratification to optimize therapeutic responses (70). Currently, there is a lack of consensus on standardized methodologies and scoring systems for assessing S100 protein expression, which hampers the integration of these markers into clinical decision-making (71). Resistance mechanisms also pose significant hurdles. For example, the ability of S100P to inactivate p53 and promote therapy-induced senescence suggests that targeting S100 proteins alone may be insufficient without combination strategies to overcome compensatory pathways (32). Furthermore, the immunosuppressive effects mediated by certain S100 proteins within the tumor microenvironment may limit the efficacy of immunotherapies unless these pathways are concurrently addressed (44). Clinical translation is additionally impeded by the limited availability of potent and selective S100 inhibitors with favorable pharmacokinetic and safety profiles. While some small molecule inhibitors and neutralizing antibodies have entered clinical trials, their therapeutic windows and long-term effects remain to be fully characterized (10). The potential for overlapping toxicities, especially when combined with other modalities such as chemotherapy, radiation, or immunotherapy, requires careful evaluation (72). Moreover, the identification of predictive biomarkers for response to S100-targeted therapies is still in its infancy. The heterogeneity of BC and the complex interplay between S100 proteins and other oncogenic pathways necessitate comprehensive biomarker panels rather than single markers to guide treatment selection (73). Integration of molecular profiling with clinical parameters is essential to overcome these challenges. In summary, while targeting S100 proteins offers a novel avenue for BC therapy, overcoming the challenges related to specificity, tumor heterogeneity, resistance, and biomarker development is critical for successful clinical application. These observations indicate that S100 proteins are promising targets for combination strategies aimed at overcoming therapy resistance in bladder cancer.

### 4.4 Future directions for translational application

Advancements in molecular characterization and therapeutic development provide a foundation for translating S100 protein targeting into clinical practice for BC. Recent years have witnessed increasing preclinical and early translational efforts to develop S100-targeted therapies. Several small-molecule inhibitors (e.g., trifluoperazine, amlexanox, niclosamide) have shown efficacy in preclinical models by disrupting S100A4, S100A13, or RAGE-mediated signaling (74). Neutralizing antibodies against S100A8/A9 have demonstrated tumor-suppressive and immune-modulatory effects in animal studies, particularly through attenuation of myeloid-derived suppressor cell recruitment. In addition, RAGE antagonists such as FPS-ZM1 and azeliragon (TTP488)—originally developed for inflammatory and neurodegenerative diseases—are being explored for potential repurposing in oncology (60). Although no S100-targeted therapy has yet entered late-stage clinical evaluation for bladder cancer, these early studies provide proof of concept that S100 inhibition can modulate tumor proliferation, invasion, and immune evasion. Ongoing clinical trials of RAGE inhibitors in other malignancies and chronic inflammatory disorders may offer critical insights and safety data to facilitate translation into bladder cancer–specific applications (75). Integrating genomic, transcriptomic, and proteomic data can facilitate the identification of patient subgroups most likely to benefit from S100-targeted therapies (76). For example, molecular subtyping of MIBC has revealed distinct profiles that may correlate with differential S100 protein expression and therapeutic vulnerabilities (71). Emerging technologies such as next-generation sequencing and liquid biopsy enable non-invasive monitoring of S100 protein-related biomarkers, potentially allowing real-time assessment of treatment response and disease progression (77).

The detection of small non-coding ribonucleic acids(RNAs) and their modifications associated with S100 protein regulation may further enhance biomarker discovery and precision medicine approaches (77). Combination therapies incorporating S100 inhibitors with established modalities, including chemotherapy, immunotherapy, and radiation, are promising strategies to enhance efficacy and overcome resistance. For instance, the immunosuppressive role of S100P suggests that its inhibition could synergize with immune checkpoint blockade to improve anti-tumor immunity (40). Clinical trials exploring such combinations are warranted to validate these approaches. Personalized medicine initiatives emphasize the need for predictive biomarkers to guide therapy selection. Protein-based biomarkers related to S100 family members, alongside other molecular markers, may refine patient stratification and optimize neoadjuvant and adjuvant treatment regimens (71, 78). The development of multimarker panels combining S100 proteins with other oncogenic and immune-related factors could improve prognostic accuracy and therapeutic decision-making (71). Furthermore, advances in drug design, including the development of highly selective small molecule inhibitors and neutralizing antibodies targeting specific S100 proteins, are critical. Structural studies elucidating the binding interfaces and conformational dynamics of S100 proteins will facilitate rational drug design and improve therapeutic specificity (59). The ongoing clinical evaluation of S100 inhibitors in other cancer types provides valuable insights that can be leveraged for BC applications (10). In addition to summarizing molecular mechanisms and preclinical evidence, we also reviewed ongoing and completed clinical trials related to BC to better illustrate the current translational landscape. This summary helps highlight research gaps and informs future clinical strategies. Collectively, these ongoing efforts underscore the translational potential of S100-targeted strategies and highlight the need for dedicated clinical evaluation in bladder cancer.

## 5 Conclusion

The S100 protein family plays a multifaceted and dynamic role in the pathogenesis and progression of BC. Through regulation of cell proliferation, migration, immune modulation, and extracellular signaling, S100 proteins contribute to tumor aggressiveness, immune evasion, and therapeutic resistance. Their aberrant expression across different tumor stages, molecular subtypes, and immune phenotypes positions them as promising biomarkers for diagnosis, prognosis, and therapeutic response prediction. Specific members such as S100C, S100A8, S100A9, and S100A13 exhibit distinct expression patterns in non-muscle-invasive and muscle-invasive disease, with functional implications for tumor behavior. Mechanistic insights further highlight the contributions of S100 proteins to epithelial-mesenchymal transition, inflammatory signaling, and immune checkpoint regulation. Although no S100-targeted therapies have yet been approved, preclinical evidence supports their potential as therapeutic targets. Small molecules (e.g., trifluoperazine, amlexanox), biologics (e.g., monoclonal antibodies), and inhibitors of S100–RAGE interactions represent promising strategies currently under investigation. Future research should focus on overcoming challenges related to functional redundancy, context-dependent effects, and clinical validation. Incorporating S100 profiling into multi-omics and precision oncology frameworks may enhance risk stratification, enable biomarker-guided therapy, and identify novel combination strategies. Overall, the S100 family represents a valuable frontier in BC research with translational potential for improving diagnosis, prognosis, and therapeutic outcomes.

## References

[1] Bresnick AR Weber DJ Zimmer DB . S100 proteins in cancer. Nat Rev Cancer. (2015) 15:96–109. doi: 10.1038/nrc3893, PMID: 25614008 PMC4369764

[2] Chen H Xu C Jin Q Liu Z . S100 protein family in human cancer. Am J Cancer Res. (2014) 4:89–115. doi: 10.3901/JME.2014.16.089, PMID: 24660101 PMC3960449

[3] Donato R . Perspectives in S-100 protein biology. Review article. Cell Calcium. (1991) 12:713–26. doi: 10.1016/0143-4160(91)90040-l, PMID: 1769063

[4] Sreejit G Flynn MC Patil M Krishnamurthy P Murphy AJ Nagareddy PR . S100 family proteins in inflammation and beyond. Adv Clin Chem. (2020) 98:173–231. doi: 10.1016/bs.acc.2020.02.006, PMID: 32564786

[5] Leśniak W . Epigenetic regulation of S100 protein expression. Clin Epigenet. (2011) 2:77–83. doi: 10.1007/s13148-011-0023-9, PMID: 21949546 PMC3156319

[6] Salama I Malone PS Mihaimeed F Jones JL . A review of the S100 proteins in cancer. Eur J Surg Oncol. (2008) 34:357–64. doi: 10.1016/j.ejso.2007.04.009, PMID: 17566693

[7] Hermani A De Servi B Medunjanin S Tessier PA Mayer D . S100A8 and S100A9 activate MAP kinase and NF-kappaB signaling pathways and trigger translocation of RAGE in human prostate cancer cells. Exp Cell Res. (2006) 312:184–97. doi: 10.1016/j.yexcr.2005.10.013, PMID: 16297907

[8] Fei F Qu J Zhang M Li Y Zhang S . S100A4 in cancer progression and metastasis: A systematic review. Oncotarget. (2017) 8:73219–39. doi: 10.18632/oncotarget.18016, PMID: 29069865 PMC5641208

[9] Mishra SK Siddique HR Saleem M . S100A4 calcium-binding protein is key player in tumor progression and metastasis: preclinical and clinical evidence. Cancer Metastasis Rev. (2012) 31:163–72. doi: 10.1007/s10555-011-9338-4, PMID: 22109080

[10] Bresnick AR . S100 proteins as therapeutic targets. Biophys Rev. (2018) 10:1617–29. doi: 10.1007/s12551-018-0471-y, PMID: 30382555 PMC6297089

[11] Dyrskjøt L Hansel DE Efstathiou JA Knowles MA Galsky MD Teoh J . Bladder cancer. Nat Rev Dis Primers. (2023) 9:58. doi: 10.1038/s41572-023-00468-9, PMID: 37884563 PMC11218610

[12] Droller MJ . Bladder cancer: state-of-the-art care. CA Cancer J Clin. (1998) 48:269–84. doi: 10.3322/canjclin.48.5.269, PMID: 9742894

[13] Guo CC Lee S Lee JG Chen H Zaleski M Choi W . Molecular profile of bladder cancer progression to clinically aggressive subtypes. Nat Rev Urol. (2024) 21:391–405. doi: 10.1038/s41585-023-00847-7, PMID: 38321289

[14] Mikhaleva LM Pechnikova VV Pshikhachev AM Rogov KA Gusniev MA Patsap OI . Bladder cancer: update on risk factors, molecular and ultrastructural patterns. Curr Med Chem. (2021) 28:8517–33. doi: 10.2174/0929867328666210309111731, PMID: 33687878

[15] Kiemeney LA Schoenberg M . Familial transitional cell carcinoma. J Urol. (1996) 156:867–72. doi: 10.1016/S0022-5347(01)65644-1 8709350

[16] Baffa R Letko J McClung C LeNoir J Vecchione A Gomella LG . Molecular genetics of bladder cancer: targets for diagnosis and therapy. J Exp Clin Cancer Res. (2006) 25:145–60., PMID: 16918124

[17] Cheng S Andrew AS Andrews PC Moore JH . Complex systems analysis of bladder cancer susceptibility reveals a role for decarboxylase activity in two genome-wide association studies. BioData Min. (2016) 9:40. doi: 10.1186/s13040-016-0119-z, PMID: 27999618 PMC5154053

[18] Khalifa MK Bakr NM Ramadan A Abd EK Desoky E Nageeb AM . Implications of targeted next-generation sequencing for bladder cancer: report of four cases. J Genet Eng Biotechnol. (2021) 19:91. doi: 10.1186/s43141-021-00182-7, PMID: 34152511 PMC8217481

[19] Guo CC Czerniak B . Bladder cancer in the genomic era. Arch Pathol Lab Med. (2019) 143:695–704. doi: 10.5858/arpa.2018-0329-RA, PMID: 30672335

[20] Chen CL Chung T Wu CC Ng KF Yu JS Tsai CH . Comparative tissue proteomics of microdissected specimens reveals novel candidate biomarkers of bladder cancer. Mol Cell Proteomics. (2015) 14:2466–78. doi: 10.1074/mcp.M115.051524, PMID: 26081836 PMC4563729

[21] Wan FC Cui YP Wu JT Wang JM Z, L. Q Gao ZL . The PPI network and cluster ONE analysis to explain the mechanism of bladder cancer. Eur Rev Med Pharmacol Sci. (2013) 17:618–23. doi: 10.1007/s13318-012-0097-6, PMID: 23543444

[22] Danishuddin Haque MA Khan S Kim JJ Ahmad K . Molecular landscape of bladder cancer: key genes, transcription factors, and drug interactions. Int J Mol Sci. (2024) 25:10997. doi: 10.3390/ijms252010997, PMID: 39456780 PMC11507096

[23] Cheng L Davison DD Adams J Lopez-Beltran A Wang L Montironi R . Biomarkers in bladder cancer: translational and clinical implications. Crit Rev Oncol Hematol. (2014) 89:73–111. doi: 10.1016/j.critrevonc.2013.08.008, PMID: 24029603

[24] Adeyelu TT Moya-Garcia AA Orengo C . Exploiting protein family and protein network data to identify novel drug targets for bladder cancer. Oncotarget. (2022) 13:105–17. doi: 10.18632/oncotarget.28175, PMID: 35035776 PMC8758182

[25] Memon AA Sorensen BS Meldgaard P Fokdal L Thykjaer T Nexo E . Down-regulation of S100C is associated with bladder cancer progression and poor survival. Clin Cancer Res. (2005) 11:606–11. doi: 10.1158/1078-0432.606.11.2, PMID: 15701847

[26] Cross SS Hamdy FC Deloulme JC Rehman I . Expression of S100 proteins in normal human tissues and common cancers using tissue microarrays: S100A6, S100A8, S100A9 and S100A11 are all overexpressed in common cancers. Histopathology. (2005) 46:256–69. doi: 10.1111/j.1365-2559.2005.02097.x, PMID: 15720411

[27] Liu J Li X Dong GL Zhang HW Chen DL Du JJ . In silico analysis and verification of S100 gene expression in gastric cancer. BMC Cancer. (2008) 8:261. doi: 10.1186/1471-2407-8-261, PMID: 18793447 PMC2567992

[28] Myers-Irvin JM Landsittel D Getzenberg RH . Use of the novel marker BLCA-1 for the detection of bladder cancer. J Urol. (2005) 174:64–8. doi: 10.1097/01.ju.0000162022.36772.a4, PMID: 15947579

[29] Orenes-Piñero E Barderas R Rico D Casal JI Gonzalez-Pisano D Navajo J . Serum and tissue profiling in bladder cancer combining protein and tissue arrays. J Proteome Res. (2010) 9:164–73. doi: 10.1021/pr900273u, PMID: 19883059

[30] Jiang L Lai YK Zhang J Wang H Lin MC He ML . Targeting S100P inhibits colon cancer growth and metastasis by Lentivirus-mediated RNA interference and proteomic analysis. Mol Med. (2011) 17:709–16. doi: 10.2119/molmed.2011.00008, PMID: 21327297 PMC3146612

[31] Whiteman HJ Weeks ME Dowen SE Barry S Timms JF Lemoine NR . The role of S100P in the invasion of pancreatic cancer cells is mediated through cytoskeletal changes and regulation of cathepsin D. Cancer Res. (2007) 67:8633–42. doi: 10.1158/0008-5472.CAN-07-0545, PMID: 17875703

[32] Gibadulinova A Pastorek M Filipcik P Radvak P Csaderova L Vojtesek B . Cancer-associated S100P protein binds and inactivates p53, permits therapy-induced senescence and supports chemoresistance. Oncotarget. (2016) 7:22508–22. doi: 10.18632/oncotarget.7999, PMID: 26967060 PMC5008377

[33] Tao J Li J Fan X Jiang C Wang Y Qin M . Unraveling the protein post-translational modification landscape: Neuroinflammation and neuronal death after stroke. Ageing Res Rev. (2024) 101:102489. doi: 10.1016/j.arr.2024.102489, PMID: 39277050

[34] Donato R Cannon BR Sorci G Riuzzi F Hsu K Weber DJ . Functions of S100 proteins. Curr Mol Med. (2013) 13:24–57. doi: 10.2174/156652413804486214 22834835 PMC3707951

[35] Grotterød I Maelandsmo GM Boye K . Signal transduction mechanisms involved in S100A4-induced activation of the transcription factor NF-kappaB. BMC Cancer. (2010) 10:241. doi: 10.1186/1471-2407-10-241, PMID: 20507646 PMC2902441

[36] Namura T Arai S Koike A Yamada S Totani M Ikemoto. M . Possible mechanism for regulation of inflammatory responses with the S100A8/A9 protein. Rinsho Byori. (2010) 58:651–7., PMID: 20715507

[37] Cui Y Li L Li Z Yin J Lane J Ji J . Dual effects of targeting S100A11 on suppressing cellular metastatic properties and sensitizing drug response in gastric cancer. Cancer Cell Int. (2021) 21:243. doi: 10.1186/s12935-021-01949-1, PMID: 33931048 PMC8086328

[38] Crew JP O’Brien TS Harris AL . Bladder cancer angiogenesis, its role in recurrence, stage progression and as a therapeutic target. Cancer Metastasis Rev. (1996) 15:221–30. doi: 10.1007/BF00437475, PMID: 8842494

[39] Yen MC Huang YC Kan JY Kuo PL Hou MF Hsu YL . S100B expression in breast cancer as a predictive marker for cancer metastasis. Int J Oncol. (2018) 52:433–40. doi: 10.3892/ijo.2017.4226, PMID: 29345293

[40] Hao W Zhang Y Dou J Cui P Zhu J . S100P as a potential biomarker for immunosuppressive microenvironment in pancreatic cancer: a bioinformatics analysis and *in vitro* study. BMC Cancer. (2023) 23:997. doi: 10.1186/s12885-023-11490-1, PMID: 37853345 PMC10585823

[41] Parodi A Traverso P Kalli F Conteduca G Tardito S Curto M . Residual tumor micro-foci and overwhelming regulatory T lymphocyte infiltration are the causes of bladder cancer recurrence. Oncotarget. (2016) 7:6424–35. doi: 10.18632/oncotarget.7024, PMID: 26824503 PMC4872724

[42] Wang W Huang G Lin H Ren L Fu L Mao X . Corrigendum: Label-free LC-MS/MS proteomics analyses reveal CLIC1 as a predictive biomarker for bladder cancer staging and prognosis. Front Oncol. (2023) 13:1216134. doi: 10.3389/fonc.2023.1216134, PMID: 38264752 PMC10803480

[43] Hilmy M Bartlett JM Underwood MA McMillan DC . The relationship between the systemic inflammatory response and survival in patients with transitional cell carcinoma of the urinary bladder. Br J Cancer. (2005) 92:625–7. doi: 10.1038/sj.bjc.6602406, PMID: 15726119 PMC2361870

[44] Huang P Wang J Yu Z Lu J Sun Z Chen Z . Redefining bladder cancer treatment: innovations in overcoming drug resistance and immune evasion. Front Immunol. (2025) 16:1537808. doi: 10.3389/fimmu.2025.1537808, PMID: 39911393 PMC11794230

[45] Miranda KJ Loeser RF Yammani RR . Sumoylation and nuclear translocation of S100A4 regulate IL-1beta-mediated production of matrix metalloproteinase-13. J Biol Chem. (2010) 285:31517–24. doi: 10.1074/jbc.M110.125898, PMID: 20685652 PMC2951226

[46] Song YY Liang D Liu DK Lin L Zhang L Yang WQ . The role of the ERK signaling pathway in promoting angiogenesis for treating ischemic diseases. Front Cell Dev Biol. (2023) 11:1164166. doi: 10.3389/fcell.2023.1164166, PMID: 37427386 PMC10325625

[47] Kim SK Kim EJ Leem SH Ha YS Kim YJ Kim WJ . Identification of S100A8-correlated genes for prediction of disease progression in non-muscle invasive bladder cancer. BMC Cancer. (2010) 10:21. doi: 10.1186/1471-2407-10-21, PMID: 20096140 PMC2828413

[B48] Nedjadi T Benabdelkamal H Albarakati N Masood A Al-Sayyad A Alfadda AA . Circulating proteomic signature for detection of biomarkers in bladder cancer patients. Sci Rep. (2020) 10:10999. doi: 10.1038/s41598-020-67929-z, PMID: 32620920 PMC7335182

[49] Hurst CD Cheng G Platt FM Castro M Marzouka NS Eriksson P . Stage-stratified molecular profiling of non-muscle-invasive bladder cancer enhances biological, clinical, and therapeutic insight. Cell Rep Med. (2021) 2:100472. doi: 10.1016/j.xcrm.2021.100472, PMID: 35028613 PMC8714941

[50] Takagi R Sakamoto E Kido JI Inagaki Y Hiroshima Y Naruishi K . S100A9 increases IL-6 and RANKL expressions through MAPKs and STAT3 signaling pathways in osteocyte-like cells. BioMed Res Int. (2020) 2020:7149408. doi: 10.1155/2020/7149408, PMID: 32149126 PMC7053464

[51] Zhang J Guo B Zhang Y Cao J Chen T . Silencing of the annexin II gene down-regulates the levels of S100A10, c-Myc, and plasmin and inhibits breast cancer cell proliferation and invasion. Saudi Med J. (2010) 31:374–81. doi: 10.1016/j.revmed.2009.09.031, PMID: 20383413

[52] Garcia V Perera YR Chazin WJ . A structural perspective on calprotectin as a ligand of receptors mediating inflammation and potential drug target. Biomolecules. (2022) 12:519. doi: 10.3390/biom12040519, PMID: 35454108 PMC9026754

[53] Robertson AG Kim J Al-Ahmadie H Bellmunt J Guo G Cherniack AD . Comprehensive molecular characterization of muscle-invasive bladder cancer. Cell. (2017) 171:540–56. doi: 10.1016/j.cell.2017.09.007, PMID: 28988769 PMC5687509

[54] Qasrawi F Meuser M Lehnhoff F Schulte M Kispert A . S100A1 expression characterizes terminally differentiated superficial cells in the urothelium of the murine bladder and ureter. Histochem Cell Biol. (2022) 158:389–99. doi: 10.1007/s00418-022-02120-1, PMID: 35648290 PMC9512885

[55] Cai Y Cheng Y Wang Z Li L Qian Z Xia W . A novel metabolic subtype with S100A7 high expression represents poor prognosis and immuno-suppressive tumor microenvironment in bladder cancer. BMC Cancer. (2023) 23:725. doi: 10.1186/s12885-023-11182-w, PMID: 37543645 PMC10403905

[56] Tanaka M Ichikawa-Tomikawa N Shishito N Nishiura K Miura T Hozumi A . Co-expression of S100A14 and S100A16 correlates with a poor prognosis in human breast cancer and promotes cancer cell invasion. BMC Cancer. (2015) 15:53. doi: 10.1186/s12885-015-1059-6, PMID: 25884418 PMC4348405

[57] Dyrskjøt L Zieger K Real FX Malats N Carrato A Hurst C . Gene expression signatures predict outcome in non-muscle-invasive bladder carcinoma: a multicenter validation study. Clin Cancer Res. (2007) 13:3545–51. doi: 10.1158/1078-0432.CCR-06-2940, PMID: 17575217

[58] Brenner AK Bruserud Ø . S100 proteins in acute myeloid leukemia. Neoplasia. (2018) 20:1175–86. doi: 10.1016/j.neo.2018.09.007, PMID: 30366122 PMC6215056

[59] Singh H Rai V Agrawal DK . Discerning the promising binding sites of S100/calgranulins and their therapeutic potential in atherosclerosis. Expert Opin Ther Pat. (2021) 31:1045–57. doi: 10.1080/13543776.2021.1937122, PMID: 34056993 PMC8551002

[60] Azizan N Suter MA Liu Y Logsdon CD . RAGE maintains high levels of NFκB and oncogenic Kras activity in pancreatic cancer. Biochem Biophys Res Commun. (2017) 493:592–7. doi: 10.1016/j.bbrc.2017.08.147, PMID: 28867179

[61] Shen L Pili R . Tasquinimod targets suppressive myeloid cells in the tumor microenvironment. Oncoimmunology. (2019) 8:e1072672. doi: 10.1080/2162402X.2015.1072672, PMID: 31646064 PMC6791429

[62] Li J Shu X Xu J Su SM Chan UI Mo L . S100A9-CXCL12 activation in BRCA1-mutant breast cancer promotes an immunosuppressive microenvironment associated with resistance to immunotherapy. Nat Commun. (2022) 13:1481. doi: 10.1038/s41467-022-29151-5, PMID: 35304461 PMC8933470

[63] Hua X Zhang H Jia J Chen S Sun Y Zhu X . Roles of S100 family members in drug resistance in tumors: Status and prospects. BioMed Pharmacother. (2020) 127:110156. doi: 10.1016/j.biopha.2020.110156, PMID: 32335300

[64] Zhou H Zhao C Shao R Xu Y Zhao W . The functions and regulatory pathways of S100A8/A9 and its receptors in cancers. Front Pharmacol. (2023) 14:1187741. doi: 10.3389/fphar.2023.1187741, PMID: 37701037 PMC10493297

[65] Huang M Wu R Chen L Peng Q Li S Zhang Y . S100A9 regulates MDSCs-mediated immune suppression via the RAGE and TLR4 signaling pathways in colorectal carcinoma. Front Immunol. (2019) 10:2243. doi: 10.3389/fimmu.2019.02243, PMID: 31620141 PMC6759487

[66] Leclerc E Fritz G Vetter SW Heizmann CW . Binding of S100 proteins to RAGE: an update. Biochim Biophys Acta. (2009) 1793:993–1007. doi: 10.1016/j.bbamcr.2008.11.016, PMID: 19121341

[67] Tarabykina S Griffiths TR Tulchinsky E Mellon JK Bronstein IB Kriajevska M . Metastasis-associated protein S100A4: spotlight on its role in cell migration. Curr Cancer Drug Targets. (2007) 7:217–28. doi: 10.2174/156800907780618329, PMID: 17504119

[68] Herwig N Belter B Wolf S Haase-Kohn C Pietzsch J . Interaction of extracellular S100A4 with RAGE prompts prometastatic activation of A375 melanoma cells. J Cell Mol Med. (2016) 20:825–35. doi: 10.1111/jcmm.12808, PMID: 26928771 PMC4831350

[69] Xia C Braunstein Z Toomey AC Zhong J Rao X . S100 proteins as an important regulator of macrophage inflammation. Front Immunol. (2017) 8:1908. doi: 10.3389/fimmu.2017.01908, PMID: 29379499 PMC5770888

[70] Bedore S van der Eerden J Boghani F Patel SJ Yassin S Aguilar K . Protein-based predictive biomarkers to personalize neoadjuvant therapy for bladder cancer-A systematic review of the current status. Int J Mol Sci. (2024) 25:9899. doi: 10.3390/ijms25189899, PMID: 39337385 PMC11432686

[71] Sanguedolce F Cormio A Bufo P Carrieri G Cormio L . Molecular markers in bladder cancer: Novel research frontiers. Crit Rev Clin Lab Sci. (2015) 52:242–55. doi: 10.3109/10408363.2015.1033610, PMID: 26053693

[72] Hasan N Yang D Gibson S Khaleghi B Ziari R Kalebasty AR . Advancements in bladder cancer treatment: The synergy of radiation and immunotherapy. Oncotarget. (2025) 16:337–46. doi: 10.18632/oncotarget.28723, PMID: 40387780 PMC12088031

[73] Kim WJ Park S Kim YJ . Biomarkers in bladder cancer: present status and perspectives. biomark Insights. (2007) 2:95–105. doi: 10.1177/117727190700200018, PMID: 19662195 PMC2717839

[74] Malashkevich VN Dulyaninova NG Ramagopal UA Liriano MA Varney KM Knight D . Phenothiazines inhibit S100A4 function by inducing protein oligomerization. Proc Natl Acad Sci U.S.A. (2010) 107:8605–10. doi: 10.1073/pnas.0913660107, PMID: 20421509 PMC2889333

[75] Bhogal I Pankaj V Roy S . Identifying RAGE inhibitors as potential therapeutics for Alzheimer’s disease via integrated in-silico approaches. Sci Rep. (2025) 15:17730. doi: 10.1038/s41598-025-01271-0, PMID: 40404684 PMC12098779

[76] Alifrangis C McGovern U Freeman A Powles T Linch M . Molecular and histopathology directed therapy for advanced bladder cancer. Nat Rev Urol. (2019) 16:465–83. doi: 10.1038/s41585-019-0208-0, PMID: 31289379

[77] Su Z Monshaugen I Klungland A Ougland R Dutta A . Characterization of novel small non-coding RNAs and their modifications in bladder cancer using an updated small RNA-seq workflow. Front Mol Biosci. (2022) 9:887686. doi: 10.3389/fmolb.2022.887686, PMID: 35923465 PMC9340255

[78] Heard JR Ahdoot M Theodorescu D Mitra AP . Biomarkers of treatment response in bladder cancer. Expert Rev Mol Diagn. (2024) 24:957–69. doi: 10.1080/14737159.2024.2428747, PMID: 39535158

